# A prospective assessment of readiness to implement an early detection of cerebral palsy pathway in a neonatal intensive care setting using the PARIHS framework

**DOI:** 10.1186/s43058-024-00581-0

**Published:** 2024-04-23

**Authors:** Amy Mulqueeney, Malcolm Battin, Ann McKillop, N. Susan Stott, Angelica Allermo-Fletcher, Sîan A. Williams

**Affiliations:** 1 Newborn Services, Starship Child Health, Te Toka Tumai – Auckland, Te Whatu Ora, Auckland, New Zealand; 2 School of Nursing, University of Auckland, Auckland, New Zealand; 3 Department of Surgery, University of Auckland, Auckland, New Zealand; 4 Neonatal Intensive Care Unit, Te Whatu Ora- Capital, Coast and Hutt Valley, Wellington, New Zealand; 5 School of Allied Health, Curtin University, Perth, Australia; 6 Liggins Institute, University of Auckland, Auckland, New Zealand; 7 Faculty of Medical and Health Sciences, Paediatrics, Child and Youth Health, University of Auckland, Auckland, New Zealand

**Keywords:** Implementation, General movements assessment, HINE, Early diagnosis, Neurodevelopment

## Abstract

**Background:**

Early detection of cerebral palsy (CP) is possible through targeted use of assessment tools. Changes in practice are needed to facilitate this shift towards earlier diagnosis of CP in New Zealand. The aim of this study was to prospectively evaluate readiness to implement an early detection of CP pathway within a level 3 neonatal intensive care unit (NICU) setting prior to any implementation taking place. The PARIHS (Promoting Action on Research Implementation in Health Services) framework was engaged to assess readiness by highlighting determinants that influence implementation outcomes as either barriers or enablers.

**Methods:**

A mixed methods approach was used. Firstly, an online staff survey assessed PARIHS sub-elements using Likert scores and free text with the intent to develop a baseline understanding of staff views. Secondly, focus groups were conducted to gain deeper understanding of barriers and enablers to implementation. Participants included health professionals involved in the first 6 months of life. Data were analysed to outline the barriers and enablers of implementation under the *Evidence* and *Context* constructs of the PARIHS framework.

**Results:**

Twenty-seven participants completed the survey, and 20 participants participated in eight focus groups and two individual interviews. Quantitative (survey) findings found 65% agreement around the usefulness of research evidence on early CP detection; however, ≤ 45% felt current resources (i.e. human, financial and IT) were sufficient for implementation. Qualitative findings (survey and focus groups) highlighted key staff concerns around resources, family impact (creating unnecessary stress), and equity (barriers to participation). Staff wanted information regarding how international evidence translates to the local context and availability of timely follow-up services. Sub-elements within the *Evidence* and *Context* constructs were rated as either mixed or low (except for *Evidence - Research*, rated as high), overall indicating that Auckland NICU is at the early stages of readiness to implement the early CP detection pathway.

**Conclusion:**

This work may resonate with other neonatal services preparing to implement CP early detection pathways. Resourcing has a major role in facilitating implementation of pathways and uncertainty about resources is a barrier to implementation. Ongoing focus on building consensus and funding is required to ensure optimal uptake.

**Supplementary Information:**

The online version contains supplementary material available at 10.1186/s43058-024-00581-0.

Contribution to the literature

- Highlights the importance of assessing the status quo, particularly context, before embarking on implementation
- Outline of staff perceptions of barriers and enablers to implementation of early diagnosis of CP in Te Toka Tumai, Auckland, NICU.
- Equity has been highlighted as a critical consideration for any implementation of change in health service delivery with staff concerns for potentially inadvertently increasing inequity.
- Findings from this study may be used to help prepare, advocate, and successfully implement changes in health service delivery to facilitate earlier detection of CP.

## What is already known on this topic

- International best practice recommendations encourage the use of key diagnostic tools to support early detection of cerebral palsy in infants meeting high risk criteria.
- The PARIHS framework is designed to support and evaluate the implementation of change in health practice, taking into consideration the context (i.e. setting) in which you intend to implement change.

## Introduction

Early detection of cerebral palsy (CP) pathways is increasingly being used worldwide [1–4] to enable earlier diagnosis, intervention, and family support [5]. Early detection increases the potential for timely targeted intervention during peak neuroplasticity and enables the provision of psychological support to families [5]. For infants with known antenatal, perinatal, or postnatal risk factors for CP, a combination of assessments is recommended ≤ 5 months of age (corrected for prematurity), including the General Movements Assessment (GMA), Hammersmith Infant Neurological Examination (HINE), and magnetic resonance imaging (MRI) [5].

In Aotearoa New Zealand (NZ), data from the NZ CP Register report the median age of first CP description to be 17 months, with only 11% of children receiving a CP diagnosis by 6 months of age [6]. Approximately 26% of all children with CP on the NZ CP Register identify as Māori [6] (the indigenous people of NZ comprising approximately 17% of the overall population), and whilst the timing (i.e. age) of first CP description is indicated to be similar for Māori and non-Māori, inequitable health outcomes and experiences for Māori across NZ are apparent. For example, a relatively higher proportion of Māori with CP are indicated to be living in geographical areas in NZ of higher socioeconomic deprivation [6, 7] regions, and there is also growing evidence of poor health outcomes (e.g. respiratory health) for Māori children with CP [8]. Co-design workshops [9] and a national family survey [10] report delays in receipt of diagnosis plus issues with communication and information provision about the CP diagnosis, indicating opportunities for improvement. Furthermore, surveys of NZ health professionals reveal variation in uptake of assessments and an absence of clear pathways for practice [11]. In national efforts to facilitate earlier detection and communication of a CP diagnosis, an early detection pathway was developed, utilising local insights and international evidence [5] (Additional file 1).

Implementing research into practice can take considerable time [12] but can be aided through the recognition of contextual, organisational, and cultural factors that may initially limit or impede change in practice. An appropriate framework or model is needed to guide the implementation of new initiatives in healthcare because up to two thirds of the evidence introduced into an organisation may fail due to poor implementation processes [13] including the lack of assessment of key factors impacting on its successful uptake [14]. Our team chose the Quality Implementation Framework (QIF) [15] for its comprehensive guide for our purposes through the steps of implementation of an early detection of CP (high risk for infants < 5 months) pathway within a level 3 neonatal intensive care unit (NICU) setting. In line with phase one of the QIF in which factors in the host setting are assessed for their impact on implementation, our team used the Promoting Action on Research Implementation in Health Services (PARIHS) framework [16] for its demonstrated usefulness to specify determinants that act as barriers and enablers influencing implementation outcomes under three constructs: Evidence, Context, and Facilitation [17, 18]. Because the PARIHS framework was used prospectively in this early stage of implementation, this article reports on the identification of the barriers and enablers determined in the constructs of *Evidence and Context* to inform ongoing planning for implementation of the early detection of CP pathway. A later development of PARIHS, (i-)PARIHS, was not chosen partly because the importance, interactions, mechanisms, and sufficiency of additional elements/sub-elements in relation to implementation outcomes are not yet firmly established [19] and also because of the experience of this research team in the use of PARHIS.

## Methods

### Study design and setting

A mixed-method, explanatory design was chosen to best identify the barriers and enablers to successful implementation in the clinical setting thereby assessing readiness to change.

Firstly, a questionnaire with quantitative and qualitative questions, adapted from a detailed description of the PARIHS constructs of Evidence and Context (Rycroft-Malone 2011) [20], was administered. Secondly, focus groups and interviews, questions, and discussion topics were framed again based on Evidence and Context, thereby providing a broader and more complete understanding of barriers and enablers than either approach alone [21]. The study was set at Te Toka Tumai Auckland NICU, an urban 45-bed level 3 NICU in NZ, and the associated Starship Children’s Hospital community and developmental paediatrics services.

All procedures for this study were approved by the Auckland Health Research Ethics Committee (approval number: 23360). Study findings are reported according to the COnsolidated criteria for REporting Qualitative research (COREQ) [22].

### Participants

All staff involved in the care of current or former infants admitted to Auckland NICU during their first 6 months of life were invited to take part. Participants were excluded if they had worked less than 6 months with the organisation. The same staff were invited to participate in both the survey and focus groups, and the numbers were limited by those who had capacity and wished to participate. At least two professionals from each group participated.

Survey recruitment occurred over a 6-week period (April–May 2022), distributed to staff (NICU medical and nursing staff plus allied health working with neonates e.g. occupational therapists, speech therapists, developmental paediatrics and community therapy services) using group email lists (~200 staff) and via study flyers. Leaders of groups not based in the NICU (developmental paediatrics and community therapists) were additionally contacted by study co-ordinator to invite their participation and explain relevance.

Focus group recruitment (October–December 2022) involved the same group as above and was completed via staff email, in addition to in-person approaches to key personnel (e.g. community developmental therapists) to ensure that specific insights from all targeted staff groups were represented by at least two participants. Recruitment continued until each of the targeted health profession groups were represented and data saturation was reached (no new themes identified). Where key informants were unavailable for focus groups, they were interviewed individually.

### Procedures

The survey was electronically distributed using an online software (Qualtrics™) via an anonymous link. Participants were advised that by completing the survey they were consenting to participate. The survey design followed the PARIHS constructs and sub-elements [20], formatted to include Likert scale questions relating to agreement with statements (strongly disagree to strongly agree), and allowed for optional free-text responses. Additional questions collected participant demographic information and explored practicalities of the pathway (i.e. using MRI, GMA, and HINE) (Additional file 2).

Semi-structured focus groups (grouped by profession to minimise power imbalance) took place in a private meeting room within the workplace in groups of 2–5 over approximately 1 h. Two participants chose to interview individually via zoom. Discussions were facilitated by a neonatal nurse (AM) familiar with the topic and the study setting. The interviewer was known to some of the participants but not in a supervisory or management role nor were they involved in the development of the early detection of CP pathway. Interviewer preparations were supported by AMc and SW. Focus group interview topics were informed by the survey results and guided the discussion. Any concerns that were raised could be given in-depth discussion to gain greater understanding. Groups were encouraged to focus on what they felt was important, and additional prompts were included for further comment (Additional file 3). All focus groups were recorded and professionally transcribed. Participants were advised in advance that they would not be able to amend the transcripts after the interview to protect the context of other participants' comments.

### Data analysis and reporting

Survey attempts were excluded if there were no responses beyond consent. The remaining results were compiled using excel with frequencies and percentage data provided for descriptive analysis. For ease of reporting, ‘strongly agree’ and ‘agree’ responses have been combined, as have ‘strongly disagree’ and ‘disagree’ responses. Free-text responses were collated and used to inform context for the focus group discussions.

Qualitative data was analysed according to the framework method [23] using the constructs of PARIHS [16], seeking to identify barriers and enablers to implementation in the Evidence and Context domains. Coding was completed (AM) with NVIVO 1.7.1 software using the PARIHS constructs as themes, with coding definitions emerging from each construct (Additional file 4). Coding was peer reviewed by another author (SW) against the defined theme definitions. Data saturation was reached when no new themes were being added.

## Results

### Participants

The survey had 27 usable responses (19 completed in full) from nurses (48%, *n* = 13), neonatologists (26%, *n* = 7), allied health professionals (occupational therapist, physiotherapist, speech therapist (15%, *n* = 4), developmental paediatricians (7%, *n* = 2), and a neonatal registrar (< 1%, *n* = 1). Of those completing a question about where they saw their patients, 64% (*n* = 16) indicated that they saw patients in the NICU, 12% (*n* = 3) in the community/as outpatients only, and 24% (*n* = 6) in both settings. Most (78%, *n* = 78) had over 10 years of experience working within child health with 11% (*n* = 3) between 4 and 10 years and 11% (*n* = 3) with 1–3 years’ experience. The 27 responses represent a response rate of just over 10%, but all relevant groups were represented.

Eight focus groups and two individual interviews were held involving 20 staff representing allied health inpatient and community, nursing, nursing leadership, neonatologists, developmental paediatricians. Two focus group participants were trained in GMA. Four focus group participants were trained in HINE. No further demographic characteristics are provided to protect confidentiality of participants.

Table 1 summarises focus group results categorised by PARIHS sub-constructs and ranked according to strength as outlined by Rycroft-Malone 2011 [20]. To reduce repetition, results for both the survey and the interviews are structured according to the constructs of the PARIHS framework.

**Table 1:** A summary or readiness to implement and corresponding ratings for the strength of each construct and sub-element of PARIHS

**PARIHS construct: evidence**		
Sub-element	Data/results from focus groups and interviews	Strength [20]
Research	• Many valued that practice recommendations were based on a systematic review published in a well-respected journal • Staff valued literature as relevant but not the only thing to consider, also valuing other ‘evidence’ sub elements • Some staff place high value on the ‘pathway’ as best practice, the value in their use for setting standards in practice, and the general value of early intervention • Some staff concerned about the quality of literature on which the recommendations were based and the reliability of assessments	**High** High-quality research is valued by staff as one important aspect of implementing change but not the only aspect Some staff are actively and critically engaged with the literature
Clinical experience	• Many staff are concerned about potential equity issues if the pathway is not able to be implemented across all regions • Staff query the need for early diagnosis in NZ given symptom-based treatment rather than diagnosis-based treatment • Mixed consensus on the research evidence that underlies the need for the early diagnosis pathway in terms of evidence for early intervention and therefore the ‘need’ for early diagnosis. Staff are concerned about providing the label of CP so early, given the ‘baggage’ associated with the label. A label of ‘at risk’ or another term may be preferable • Concern that ‘other’ conditions may be overlooked if the focus is on CP • Clinicians across disciplines see benefit from providing consistent assessments with clear criteria and outcomes • Clinical experience values professionals providing consistent information to parents	**Mixed** Staff see benefit of providing consistent assessments and information to families but feel uncomfortable with giving CP diagnosis at 3 months Good consensus within allied health although they state that regionally there is no consensus No currently consensus amongst doctors around need/importance of early diagnosis of CP
Family experience	• Taboo label of CP, with low knowledge about CP for most families • Staff concerned pathway (i.e. MRI, GMA, discussion about CP) might further increase anxiety in NICU families • Staff would like to know the effects of pathway on families who do not go on to have CP diagnosis • Staff value how this pathway would impact families who come to have a CP diagnosis • Current system does not have a clear pathway and can lead to mistrust in health services if families feel a diagnosis has been kept from them	**Mixed** High: family experience valued as evidence, family integrated care model values partnership with families, family viewpoint highly relevant Low: families not involved in this study
Local data	• Only a small proportion of (NZ) infants meeting criteria for assessment pathway develop CP • Lack of data collection of CP diagnosis by hospital • Current service needs based not diagnosis based • Staff noticing more conversations about the brain and long-term developmental outcomes	Lack of systematic methods for collection and analysis of local data
**PARiHS construct: context**		
Resources	• Lack of resources both staffing and systems (e.g. MRI availability, IT systems, clinic times, waiting lists) • Low FTE for allied health • This study seen as an opportunity to potentially bring resources • Skilled and motivated staff, including staff being trained in GMA	**Low** Current resources perceived to be inadequate (i.e. staffing, systems, MRI availability)
Culture	• Low awareness of the pathway, especially amongst nurses • Need to improve communication between professional groups • Staff value improving equity in healthcare and are concerned guideline could inadvertently increase inequity • Currently the allied health role is positioned as a ‘visitor’ to the NICU • Desire to provide gold standard care, if it meets the needs of our population • Mutual respect and collegiality between professions	**Mixed** Low: low awareness of early diagnosis of CP amongst inpatient staff Culture has not had the chance to embrace/reject due to lack of awareness High: enthusiasm for providing neuroprotective care, understanding GMA and HINE could be complementary Staff value providing equitable healthcare
Leadership	• NICU leaders unwilling to support if resources are not in place • Many competing priorities • Leaders willing to engage and find out more • Nursing leadership have established staff education pathways and ways of capturing time use • Support from nurse educators for access to knowledge and training • Leaders from different professional groups have great respect for each other • Staff unsure/disagreed that the wider organisation supported innovation and innovative people	**Low** Leadership support is currently low—primarily due to limited resources NICU leaders open to implementation if financial and staffing resources can be sustainably provided across inpatient and community. Leadership would like more information on the cultural fit of the pathway and are concerned for possible equity issues
Evaluation	• Staff very interested in how families will find this process • Some existing evaluation already in place; however, rate of CP diagnosis is not captured by hospital system • Systems already in place to capture inpatient nursing time use and could capture any increase in workload • Meaningful qualitative evaluations with families are very labour intensive	**Low** CP diagnosis not centrally reported by hospital staff Meaningful qualitative research on family experience is resource intensive The voluntary NZ CP Register will allow future evaluation (albeit with a time lag)

*FTE*, full-time equivalent hours of employment; *IT*, information technology

### PARIHS construct: evidence

There are four ‘sub-elements’ within the Evidence construct of the framework: *Research evidence*, *Clinical experience*, *Family experience*, and *Local data*, with ratings summarised in Table 1.

#### ‘Research evidence’ as evidence

**Fig. 1:**
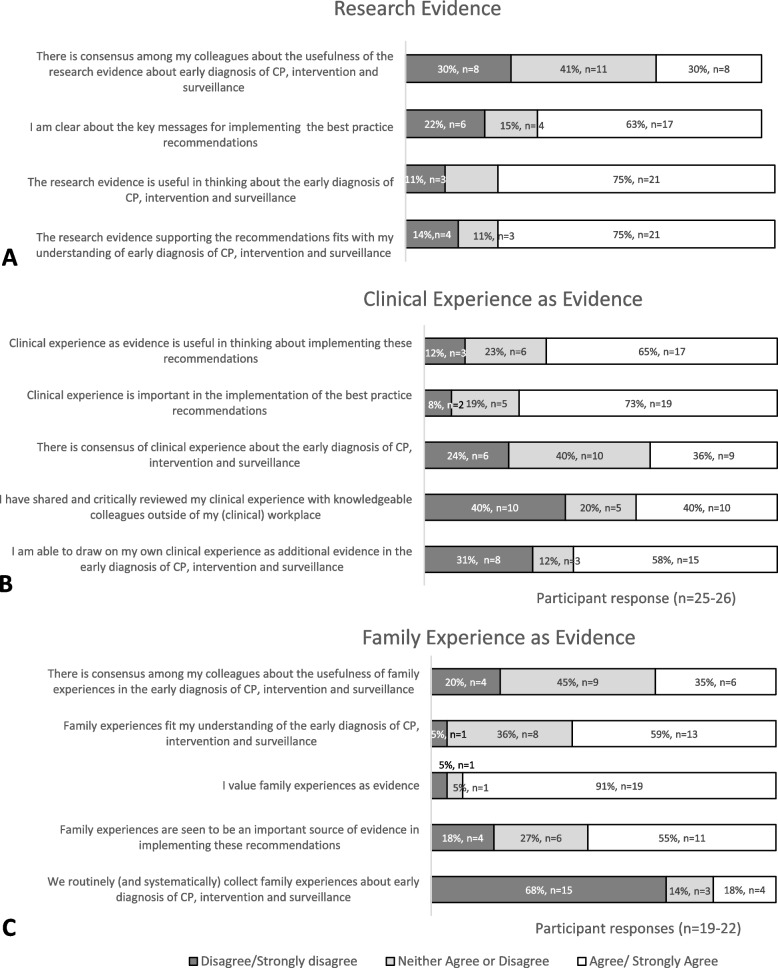
Survey responses to statements regarding **A** research as evidence, **B** clinical experience as evidence, and **C** family experience as evidence

In the survey, there was high (77.8%, *n* = 21/27) agreement (i.e. those responding agree/strongly agree) to statements relating to the evidence being useful in thinking about early diagnosis of CP and that the evidence supporting the recommendations was a fit for respondents’ own understanding of early diagnosis. Only 30% (*n* = 8/27) agreed there was consensus about the usefulness of the research evidence surrounding early diagnosis of CP (Fig. 1A). Two participants stated within the free text that there were ‘*major errors in the guideline*’, with one further questioning the ‘*low positive predictive values*’ of the assessments. One commented within this section that there was no support ‘*on a practical level*’ given that there were no apparent plans for increasing the number of staff hours allocated by funding.

Qualitative data were themed as barriers and enablers regarding ‘Importance of the evidence’ (included comments indicating approval or controversy about guideline recommendations or evidence base), ‘Consensus’, and ‘Evidence for treatment options’.

The theme ‘Importance of the evidence’ (i.e. the value staff placed on the guideline and evidence behind the guideline) was discussed in all groups. The following quotes indicate concerns about the strength of evidence for the pathway and the accuracy of MRI for diagnosis:

‘There do seem to be some trials coming, bigger trials, but not published yet. So, I believe this, you know, is a bit premature coming out before we actually have the evidence to show that it works.’ -S7, P19

‘While the sensitivity and the specificity of the MRI and the Hammersmith are high, their positive predictive value is not. So ... there’s reasonable chance that if you say someone has cerebral palsy they don’t actually.’ -S7, P19

Queries were raised whether appropriate interventions/management were already being provided in practice, as such, the potential ‘harm’ of not labelling a baby as CP is minimal in NZ:

‘In terms of actual therapy input, you shouldn’t be dependent on a diagnosis.’ -S1, P1

A further barrier was noted in a comment that three months seemed too early to diagnose and/or exclude a diagnosis of CP.

‘Severe is fine, I’m very happy to make that call. But under six months, you know, unless they’re severe, then, I start to talk about, well, risk of or, you know, maybe or needing more information. Very uncomfortable around making a definitive diagnosis at that stage.’ -S6, P16

Enablers regarding research evidence were identified from all professional groups, with the majority coming from community allied health focus groups. Two of the most frequently occurring enablers included comments that the assessment pathway added robustness and consistency to neurological assessments and that it also allowed allied health to provide honest communication and clear consistent education to families in the community, such as:

‘Feeling really positive about doing something that we know and we recognise as being a gold standard practice, and I think we want to do it well’ -S1, P1

‘But just having a pathway like this where you have those, you know, [CP is] mentioned numerous times and you have those points of contact where it’s going to be discussed again and again [with the family]’ -S4, P12

Consensus about the research evidence was represented across six groups by 15 barrier and 11 enabler quotes. Reasons for lack of consensus focused on whether early diagnosis of CP would impact on long-term outcomes for infants, for example:

‘There’s concern about it not being extremely child centred, about it putting the impairment at the centre rather than the child and the family. And that these sorts of guidelines where generated, .. [came from] countries where you need a diagnosis to get intervention’. -S4, P11

‘There is lots of things impacting on care but I’m not sure that not having an early diagnosis is, you know, the most important one of those factors’ -S3, P9

‘I haven’t seen any evidence yet that has convinced me that early intervention makes any difference to the outcome, so I don’t believe that a delayed diagnosis makes any difference to outcome’. -S7, P19

Some participants considered the usefulness of the CP pathway was dependent on the availability of, and evidence for, early treatment options that if an early diagnosis was to be given, there needed to be some evidence-based, effective, therapy and support on offer. Examples of this include:

‘If you’re going to be picking up these children you’ve got to know that you’ve got interventions that are going to have a meaningful, clinically significant impact on how they turn out’. -S3, P9

‘Looking at the different evidence, a lot of the things were around supporting babies sleep, supporting maternal mental health, you know, all of these type of more general things’. -S6, P16

Some participants agreed with the intent of the pathway in that early intervention has potential benefits and that it would encourage parents to be more attentive to neurodevelopment, for example:

‘I know that you want to use the plasticity of the brain so that definitely the early intervention has promise.’ -S5, P15

#### ‘Clinical experience’ as evidence

In the survey, agreement was high (> 65%, *n* = 17/26) that clinical experience as evidence is useful and important for implementation, but consensus was mixed about what is known from clinical experience about early diagnosis of CP intervention and surveillance (Fig. 1B).

Clinical experience was discussed in all *groups and interviews*, with 52 barrier and 23 enabler-related comments. There was concern for labelling an infant so early with CP given the ‘baggage’ associated with this, with some clinicians feeling more comfortable with ‘at risk of CP’. Some participants were concerned that other conditions might be missed with the focus of CP or that a result of low risk for CP might provide false reassurance about neurological development. Comments include:

‘I think the word cerebral palsy comes with a huge amount of baggage and I think we have to be really cautious and careful with how we use that’ -S6, P17

‘I haven’t made the diagnosis of cerebral palsy, but I’ve made sure they’d be referred to community physios …’-S6, P16:

‘They’re getting the services without that name. I tend to work, you know, what does this child and this family need in terms of services?’ -S6, P18

Participants could see the benefit of having a consistent assessment pathway where there are specific timepoints for assessments and communicating with families.

‘What early diagnosis might change in terms of this pathway, is that we’d have robust checking points’ S4, P11

Community therapy participants had clinical experience of providing therapy for children who had not yet had a diagnosis of CP from their medical team and felt they were limited in the information and education they could provide to families. Participants said this felt dishonest and that it could lead to the families not trusting them, in that:

‘It’s quite challenging doing a therapy when you can see that they’ve got hemiplegia and it’s still not a diagnosis.’-S4, P13

I feel like because there’s [currently] no clear pathway it’s very hard as a clinician in the community to sort of start broaching the subject of CP. Like at the moment, I feel like you kind of don’t want to say anything about CP until the medical team have brought it up almost. Because you don’t want the family to kind of go in and then for the medical team to go oh no, it’s fine…it kind of impacts your relationship with the family. -S4, P11

Some participants also voiced that the pathway would formalise care already being given and provide robust assessment checkpoints.

#### ‘Family experience’ as evidence

The PARIHS framework [16] defines this construct as ‘patient or client’ experience; however, this has been adjusted to ‘family’ experience given our patient group. It is important to emphasise that data collected here are the impressions of participants (staff) on families’ experiences rather than the families reporting their own experiences.

Almost all survey respondents (91%, *n* = 19/22) valued family experience as evidence, but only 35% (*n* = 6/19) felt there was consensus about the usefulness of family experiences in the early diagnosis of CP (Fig. 1C).

Family experience was discussed in all groups and interviews. Sixty-one comments were themed to ‘family experience as evidence’ being a barrier, with 35 themed as enabler. Participants discussed the diversity of cultures, regions, socio-economic status, and different ways of viewing disability amongst the families they work with. Key areas of concern were low knowledge, myths, taboos, and misconceptions about CP and that the pathway could cause unnecessary stress when parents of NICU infants are already very anxious. Comments indicated concern about when CP should be mentioned to families:

‘I feel anxious for parents who, say they had a 24 weeker, they’re generally told, if the head ultrasound is good, this is not an ultimate indication that your baby will be fine but it’s a good sign. And so cerebral palsy isn’t brought up in a lot of meetings unless there are specific, major concerns. So for the average cluster cerebral palsy’s not talked about. So for that to be popped in a meeting, they are going to freak out.’ -S2, P3

Participants indicated they would like more information about the effects of early CP assessments in families who do not go on to have a CP diagnosis. The balance between assessing what families are ready to hear and medical paternalism was a real concern:

‘You’re causing them a lot of stress and anxiety and then for them not to have cerebral palsy. But in some ways, that’s a bit of a paternalistic attitude’ -S7, P19

**Fig. 2:**
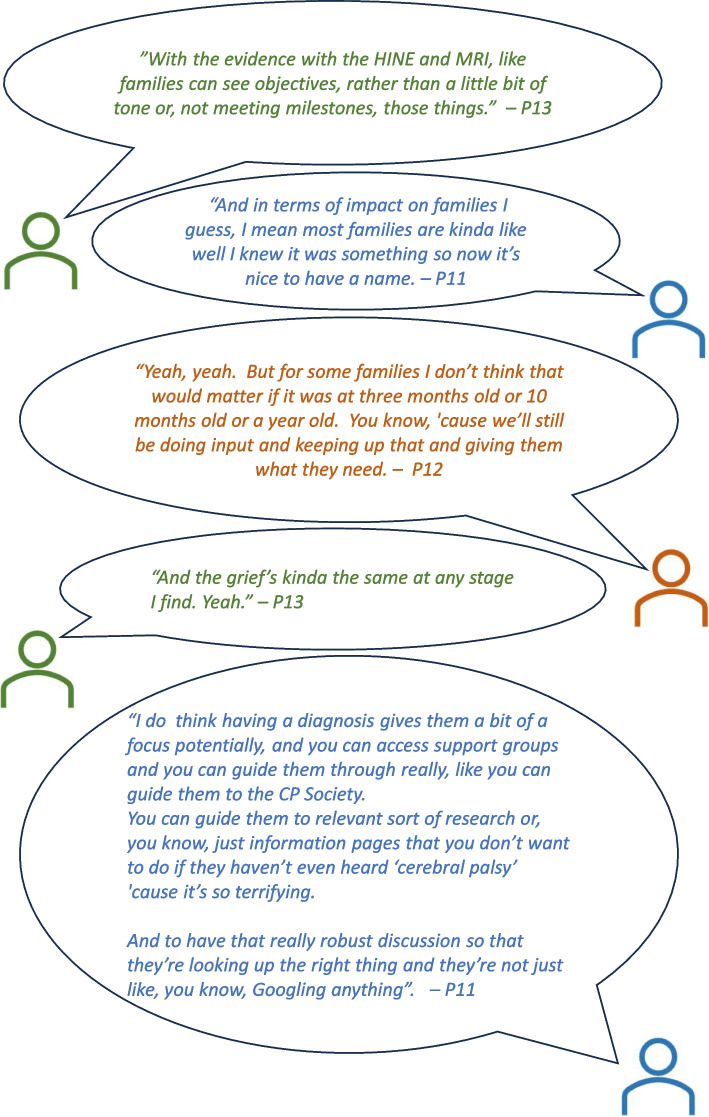
Snapshot of a conversation within the focus group regarding the impact on families

Participants could see the benefit of having a clear assessment pathway in that it would bring clarity and transparency for families (Fig. 2): ‘Sharing information is the keystone to having parents integrated in care’ (survey comment). Some participants raised concerns about the current approach to CP diagnosis leading to dissatisfaction and mistrust in health professionals, noting that some families will have a relationship with community paediatric services for up to 18 years and that ‘trust in the system might already be damaged before they start’ (S5, P15).

#### ‘Local data’ as evidence

Low prior knowledge about the early diagnosis of CP pathway prior to this study meant very limited local data were available. The pathway represented a big change of practice, and because we were assessing the status quo prior to implementation, the assessment of local data was not included in the survey.

There was little comment about local data as evidence (defined as information about CP incidence at Auckland NICU and community services) other than one group that noted only a small proportion of infants meeting criteria for the assessment pathway are likely to go on to have CP and that the current service is needs-based not diagnosis-based.

‘In terms of actual intervention therapy, it won’t change.’ -S4, P11

Local data was thought important for putting the pathway into context for local practice:

‘I think we should always look at the evidence for ourselves, because just because something’s working in Australia doesn’t mean it will work the same for us here.’ -S3, P9

### PARIHS construct: Context

The construct of Context refers to the environment or setting in which the proposed change is to be implemented and includes the sub-elements of *Receptive Context* (i.e. resources), *Culture*, *Leadership*, and *Evaluation*.

#### Context: receptive

**Fig. 3:**
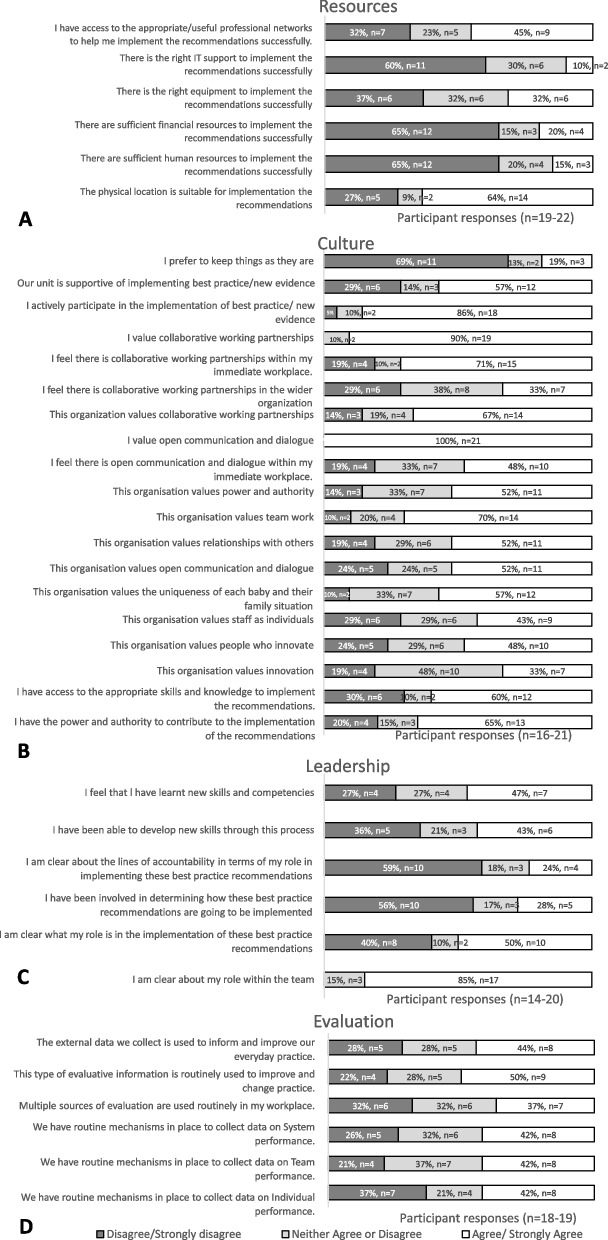
Survey responses to statements regarding the context of implementation, in relation to sub-elements: **A** Receptive (i.e. resources), **B** Culture, **C** Leadership, and **D** Evaluation

Survey responses indicated that 64% (*n* = 14/21) agreed that the physical environment was suitable for implementing early diagnosis of CP, but ≤ 45% indicated that resources (i.e. human (15%, *n* = 3/19), financial (20%, *n* = 4/19), and IT (10%, *n* = 2/19) sufficient for implementation (Fig. 3A).

‘I do not see currently… that we have any of the resources (human and non-human) that are required for the implementation of this pathway, and I am not aware of efforts to plan ahead for this’ -Survey participant

Resources were passionately discussed in all groups and interviews in relation to system and personnel resources, with 149 barrier quotes and 53 enabler quotes. Systems barriers included lack of MRI availability, current community waitlists being longer than 12 weeks (i.e. missing the GMA window), and the size of GMA videos for emailing on hospital systems. Systems enablers include an established neonatal outpatient clinic at 12 weeks which most of these infants would already qualify for and that the phones/iPads needed for filming GMA were already available.

Personnel barriers included staff already feeling that they are at capacity and mindful about extra responsibilities. In particular, staffing for allied health would need to be significantly increased in order to provide the pathway. For example:

‘We have to be mindful of that clinical creep, that’s how everything happens in nursing and nursing picks up more and more and more’. -S8, P20

Personnel enablers related to the collegiality and motivation of staff in making changes they saw to be beneficial.

‘We can say this is best practice, we’re not achieving it and we need two more staff’. -S3, P8

‘The bottom line is we will not be able to do this with the current resources we have’. -S3, P8

Indication that resources for early intervention were reported as not available/not equipped to offer early intervention (including limited psychosocial support and counselling services) once an early diagnosis is provided include:

‘Once you’ve diagnosed, what benefit is there for the family? Is the country prepared to put in some extra resourcing for these families?’ -S8, P20

There was also a concern for equity issues, with participants noting barriers for some low-income families to engage with the pathway due to extra costs. Though NZ has a public healthcare system, costs were mentioned such as transport to and from appointments, parking, and data/access to Wi-Fi for uploading videos as well as the ability to prioritise the participation in the assessments when there are significant other life stressors. A comment regarding uploading a GMA video explains that:

‘A lot of families we see don’t have data on their phone…Some families would be great, would be fine and do it easily, and some families would have difficulty.’ -S4, P12

Participants gave examples of possible solutions such as funding home visits, loaning iPads, or providing data vouchers to families in remote areas, noting that staff have gained experience with telemedicine and creative solutions through the COVID-19 pandemic.

#### Context: culture

Survey data (Fig. 3B) indicate most participants are open to change (19%, *n* = 3/16 preferring to keep things as they are), with most (57%, *n* = 12/21) perceiving that the NICU was generally supportive of implementing new evidence into practice. Generally, most participants (52–70%) agreed that the organisation valued collaboration, relationships, communication, and teamwork, with the lowest agreement for the organisation valuing innovation (33%, *n* = 7/21).

Survey data, focus groups, and interviews picked up a disconnect between nursing/medical staff and allied health. Due to current low staffing and offices located outside of NICU, allied health staff are put in the position of ‘visitor’ to NICU. Whilst there is great mutual respect, communication between the two groups could be improved, as could links between hospital care and community.

‘Bringing about practice change even with overwhelming evidence for its benefits can also be tough if there are strongly opinionated staff members who prefer the status quo or do not use best practice as the basis for their clinical care because historically things have been done differently.’ -Survey response

Focus groups and interviews reinforced the survey findings revealing low knowledge around the pathway due to limited communication (leading to limited opportunities to embrace or reject the pathway), strong mutual respect between professions, and an enthusiasm for excellence and providing the best care possible.

‘My biggest concern about this is it’s done well when we do it.’ -S1, P1

There is also high enthusiasm for providing neuroprotective care, some suggesting that early diagnosis of CP assessments could be a complementary approach.

‘I feel like these babies are the most vulnerable from being in a non-neurodevelopmental setting in terms of they’re already the quite fragile ones that are around. But it’s still quite assessment based as a protocol so it fits, but it sits alongside, would be my first thought.’ -S1, P2

‘I think it’s imperative. I think it’s where we have to go. I think it fits with the other things we’re doing,’ -S5, P14

#### Context: leadership

The survey showed low levels of agreement relating to their role in the implementation of the early diagnosis of the CP pathway (50%, *n* = 10/20) and associated lines of accountability for their role (24%, *n* = 4/17). Only 28% (*n* = 5/18) felt they had been involved in how the pathway would be implemented (Fig. 3C).

For focus group participants, the role of leadership (including champions) for implementation of the pathway was discussed as both a barrier (5 sessions, 10 comments) and enabler (5 sessions, 12 comments).

‘I’ve seen it work but you have to have a really passionate person at the top and team at the top talking about brain care all the time.’ -S2, P5

Participants in leadership roles indicated that their support for the pathway was dependent on availability of adequate resources, that they would like more information on how the pathway would impact our unique population, and that careful reflection would be needed for whether pathway implementation may inadvertently increase inequity.

‘So, right now we’re probably in a really poor place to start this. So, it’s really thinking about how we can actually advocate for those resources’ -S8, P20

‘If we don’t make an effort to make this whole thing equitable then it won’t be. And we’ve already seen that Māori and Pasifika have lower chances of surviving extreme preterm birth’ -S7, P19

‘We are super mindful of the equity issue here of this level of service being provided to the NICU babies without that commensurate path for the other babies.’ -S6, P16

#### Context: evaluation

Agreement relating to effective evaluation practices ranged between 37 and 50% (*n* = 7–9/18–19) within the survey (Fig. 3D), with barriers (3 sessions, 14 comments) and one enabler (1 comment) discussed within groups and interviews. Participants expressed interest in capturing the family experience (via qualitative interviews not ‘smiley face evaluations’) of the assessment pathway and felt this would be important in ensuring equitable rollout.

### Summary of readiness to implement early diagnosis of CP pathway

Readiness to implement is currently rated overall as low due to the large number of barriers still to be overcome to roll out this pathway, with the most obvious barrier being resource deficit (staffing, time available, finances, MRI availability). Most subconstructs are rated as mixed or low (Table 1). Evidence-research is an exception—rated as *high* as staff showed high levels of critical engagement with the literature. This means that although there was variation in levels of acceptance of the research around early diagnosis of CP and early intervention, staff valued research and saw it as an important part of the picture but not the only type of evidence.

### Practical considerations for the pathway

**Fig. 4:**
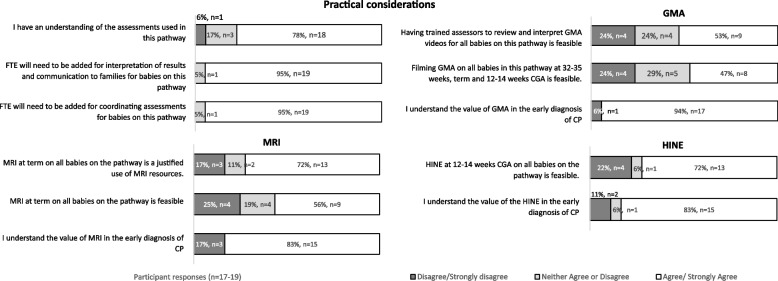
Survey responses to practical considerations of the early detection of CP pathway

Survey responses indicated high agreement (> 83%) for the value of MRI (83%, *n* = 15/18), GMA (94%, *n* = 17/18), and HINE (83%, *n* = 15/18) in the early diagnosis of CP and reasonable levels of agreement for feasibility in their use (MRI:56% *n* = 9/17, GMA:47% *n* = 8/17, HINE:72% *n* = 13/18) (Fig. 4). Almost three quarters of the respondents agreed that use of MRI at term for babies on the pathway was a justified use of resources.

Within the interviews, participants made suggestions around what the pathway could be called, what families might need, what education staff would need, and extra clinic time required to complete assessments.

## Discussion

The findings from this pre-implementation study indicate that Auckland NICU is at the early stages of readiness to implement the early detection of CP pathway, with sub-elements within the Evidence and Context constructs of the PARIHS framework rated as either mixed or low (Table 1). This work raises several critical considerations likely to resonate with health services with comments centred on issues relating to resources, the need, and consequences of a CP diagnosis (including early intervention) and equity. Of those participants expressing hesitation to implement the pathway, in the context of competing priorities and limited resources, a general impression was to delay implementing any changes so that ‘when we do it, we will do it well’. The conflict within an approach of waiting until all aspects of the pathway can be met to a high standard sits within the risk of contributing to greater disparities in practice and dissatisfaction by families and health professionals. The value of assessing readiness to implement before embarking on such a change is that findings may be used to prepare, advocate, and, ultimately, successfully implement changes in health service delivery, such as facilitating earlier detection of CP.

In line with previous research [11, 24], the issue of balancing limited resources (e.g. human, financial, MRI) with competing clinical demands is a major factor for progressing with implementation. A critical factor is the limited staffing for allied health within the NICU services, which would require either an increase in staffing or re-deployment of current staff towards early detection. Despite a growing body of evidence in support of early intervention [25–27], some health professionals queried the strength of the current evidence and highlighted concern over availability of therapy and support options for families to access. It was also noted that in NZ, a diagnosis is not needed for intervention. However, this viewpoint overlooks additional funding and opportunities afforded by a diagnosis available outside of the healthcare system (e.g. not for profit organisations working with people affected by CP). Some participants suggested refinement of MRI criteria in the pathway, as this is a significant burden on resources plus some may feel ‘obliged’ to do an MRI despite perceiving that this would not make a difference to diagnosis. This illustrates that evidence and contextual arms are linked [19]. Early CP detection is possible without MRI, which may not be safe or affordable in some circumstances. Indeed, protocols for early detection in CP within low-resource settings focus on GMA and HINE [28, 29]. Resource-related discussions also recognised that only a small proportion of infants entering the pathway will, statistically, have a final diagnosis of CP. Within the current study, health professionals raised concerns that assessments could increase anxiety both in families who have an eventual diagnosis of CP and those who go through the assessments without a diagnosis of CP. The opposite outcome could also be argued, in that assessments could be reassuring to families (as reporting related to neuroimaging [30]), and, as consistently indicated in data collected from family experiences, an earlier diagnosis is preferred [10, 31]. This sentiment also applies to the label/use of the word ‘cerebral palsy’ along this pathway; although health professionals were conscious of causing anxiety, it could be argued that using the pathway will open lines of communication and information sharing about CP from trusted health professionals (rather than families self-seeking information). Health professionals valued their clinical experience and the experience of families as evidence and would like more information on the impact of the assessments on families who do not have CP given that this will apply to most of families assessed.

The implementation of the early detection pathway for CP raised important discussions surrounding equity, particularly concerning the application for families from lower socio-economic backgrounds or those residing in greater geographical distances from services. Notably, in NZ, Māori are overrepresented in these groups, emphasising the significance of addressing this issue [32]. Health professionals participating in this study raised potential equity concerns in both contextual and cultural aspects as well as clinical and family experience as evidence. Culturally, the issue of data sovereignty was raised concerning GMA videos for Māori families, with questions surrounding the ownership and storage of these videos, emphasising the need to respect and uphold Māori cultural values and practices [33]. The ability for families to access cultural support throughout the assessment process was highlighted as an essential factor in ensuring a holistic and culturally appropriate approach to care. By integrating equity-focused strategies, healthcare providers can work towards minimising disparities and optimising the benefits of early detection for all families [34]. Equity is a current global issue, particularly in countries with a colonial history where the intergenerational health impacts to first nations people have led to poorer outcomes [35]. The PARIHS framework may benefit from embedding equity assessment when planning and facilitating implementation to reduce the risk of new interventions inadvertently increasing inequity. As outlined in Table 1 (several places) and demonstrated by the two quotes (under Context: leadership’ section), this topic is frequently raised by using PARHIS.

## Limitations

Low survey response rates could be attributed to low staff awareness around the topic of early diagnosis of CP (therefore the survey seemed less relevant). In addition, the survey design attempted to include as many of the elements adapted from Rycroft-Malone [20] as possible. Therefore, the survey length may also have put some staff off completion. Study engagement (survey response, availability/willingness to complete interview) was hampered by COVID-19 outbreaks, high unit workload, and general fatigue of health staff. On reflection, improvements could be made to the survey design to reduce its length (given participants also needed to also review the pathway/additional documents) and improve the clarity of some of the included statements. Self-reported ethnicity of participants was collected, but not reported to protect participant confidentiality, given that a relatively small number of staff working within the study setting identify as Māori. Data collection for this study did not involve the perspective of families; however, members of the research team have completed complimentary research evaluating the family perspective in NZ [9, 10], including co-design workshops held with Māori family participants focused on early health service delivery around diagnosis of CP. Findings from both studies present evidence that families are in favour of early detection of CP in NZ. Members of the team continue to be involved in ongoing research studies capturing these critical insights.

## Conclusion

This project offers promise for implementing an early detection pathway for CP within the Auckland NICU service. To ensure its success, adequate funding and system support are essential for both the diagnostic pathway and ongoing therapies, as well as psychosocial support for families. The study identified bi-directional associations between Context and Evidence, with resource-related challenges being a common factor across all elements. Future efforts will include prioritisation of family engagement in the development of new care pathways and plan to capture their experiences through surveys and interviews or co-design workshops.

While barriers were found across the PARIHS constructs, several significant enablers emerged, indicating that most staff would be willing to participate in a service that offers early diagnosis if it is well-resourced and empowering for families. Key needs for successful implementation include incorporating the family voice, securing buy-in from management, and obtaining funding for assessments and follow-up support. By addressing these aspects, the implementation of the early detection pathway can lead to improved outcomes for children and their families.

### Supplementary Information

**Additional file 1.** Early detection of cerebral palsy < 5 months—pathway.

**Additional file 2.** Early detection of cerebral palsy—readiness to implement survey.

**Additional file 3.** Focus group structure.

**Additional file 4.** Code book.

**Additional file 5.**

## Data Availability

The datasets used and/or analysed during the current study are available (de-identified) from the corresponding author on reasonable request.

## Abbreviations

CP: Cerebral palsy
GMA: General movements assessment
HINE: Hammersmith infant neurological examination
NZ: New Zealand
PARiHS: The promoting action on research implementation in health services framework
NICU: Neonatal intensive care unit
COREQ: COnsolidated criteria for REporting Qualitative research
FTE: Full-time equivalent
IT: Information technology
