# Supplementary material for: A prospective assessment of readiness to implement an early detection of cerebral palsy pathway in a neonatal intensive care setting using the PARIHS framework
Source: Implement Sci Commun. 2024 Apr 23;5:46. doi: 10.1186/s43058-024-00581-0 (PMC11036598; doi:10.1186/s43058-024-00581-0)

**Supplementary 1**: Details of the “Early detection of CP pathway (<5 months)” referred to within the study, including (A) criteria for inclusion into the pathway, and (B) a flow chart outlining the recommended timing of assessments. The recommendations were produced by the New Zealand Cerebral Palsy Clinical Network, a network within the New Zealand Child and Youth Clinical Network.

Note: for access to the most recent versions of the recommendations, visit: <https://starship.org.nz/guidelines/early-diagnosis-of-cerebral-palsy-cp-intervention-and-surveillance/>

1.A

| Criteria for inclusion within the pathways for infants admitted to NICU/SCBU |
| --- |
| 1. Infant born before 30 + 0 weeks gestation   OR   1. Infant born after 30 + 0 weeks gestation with one or more detectable risk factors for cerebral palsy    1. Weight < 1000g    2. **Intrauterine growth restriction**. Birth weight < 3rd percentile on population-based or customised growth charts or at clinician's discretion based on concern about pathological growth restriction.    3. **Abnormal findings on neuroimaging associated with CP**. (eg grade 3 and 4 intraventricular haemorrhage, post haemorrhagic ventricular dilatation, hydrocephalus, periventricular leukomalacia (PVL), stroke, brain maldevelopment).    4. **Hypoxic ischaemic encephalopathy (HIE)** - Grade 2 or 3    5. **Neonatal encephalopathy** of other aetiology    6. **Neonatal meningitis/encephalitis** - bacterial or viral    7. **Cardiac surgery**    8. **Clinical or parental concerns** or other significant risk factor - clinician discretion. |

1.B


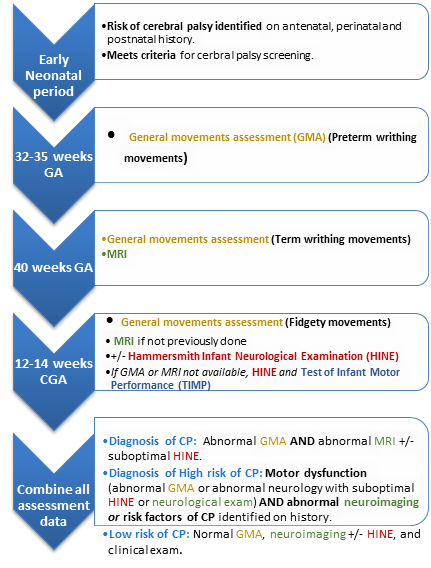

Supplement: Supplementary file 1 — Additional file 1. Early detection of cerebral palsy < 5 months—pathway. [file 43058_2024_581_MOESM1_ESM.docx]
