# Supplementary material for: A prospective assessment of readiness to implement an early detection of cerebral palsy pathway in a neonatal intensive care setting using the PARIHS framework
Source: Implement Sci Commun. 2024 Apr 23;5:46. doi: 10.1186/s43058-024-00581-0 (PMC11036598; doi:10.1186/s43058-024-00581-0)
Supplement: Supplementary file 2 — Additional file 2. Early detection of cerebral palsy—readiness to implement survey. [file 43058_2024_581_MOESM2_ESM.docx]

Supplementary 2: Assessment of Readiness to Implement Cerebral Palsy Early Identification Pathway Survey outline

Assessment of Readiness to Implement Cerebral Palsy Early Identification Pathway

Please begin this survey after you have reviewed the Early Identification of Cerebral Palsy Pathway (Link)

We appreciate that your time is valuable. This survey is targeted towards all health professionals working within the NICU, and health professionals working closely with children up to 6 months post discharge from the NICU.

The information you provide is anonymous and will be used to guide the implementation of the Cerebral Palsy Early Identification Pathway into our practice in a way that provides excellence in care for our NICU babies and their Whānau, and is also practical and user friendly for staff. This will enable us to provide earlier diagnosis of cerebral palsy, or detection of infants who may be at high risk for cerebral palsy, which will lead to better outcomes for the affected babies and their families.

Q1.

- I consent to take part in this study.

# BLOCK 1: A little bit about you

Q2 What is your professional background / qualifications?

- Neonatologist
- Developmental Paediatrician
- Paediatrician (Other) ________________________________________________
- Radiologist
- Neonatal Registrar
- NICU Nurse NP/NSANP
- NICU Nurse Senior/ level 4 (role optional) ____________________________________
- NICU Nurse level 3
- NICU Nurse level 2
- NICU Nurse NETP
- NICU Homecare
- Nursing Bureau
- Starship Community Nurse
- Nursing Other (role optional) ___________________________________________
- Physiotherapist
- Occupational Therapist
- Speech and Language Therapist
- Neurodevelopmental Therapist
- Dietician
- Social Worker
- Allied Health Other ________________________________________________
- Radiographer/sonographer
- Other ________________________________________________

Q3 Ethnicity (Optional)

- NZ European
- Māori
- Samoan
- Cook Island Māori
- Tongan
- Niuean
- Chinese
- Indian
- Other (Please state) ________________________________________________

Q4 Where do you see patients?

- NICU only
- Community/outpatient only
- Both inpatient and community/outpatient

Q5 Approximately how many years have you worked within child health?

- Less than 1 year
- 1-3 years
- 4-10 years
- 10+ years

Q6 Approximately how many years have worked in your current role?

- Less than 1 year
- 1-3 years
- 4-10 years
- 10+ years

# BLOCK 2: Evidence

Q7 Evidence **This section is about the Research Evidence supporting the newly released best practice recommendations for the early diagnosis of cerebral palsy (CP), intervention and surveillance.**

To what extent do you agree with the following statements.

|  | Strongly disagree | Somewhat disagree | Neither agree nor disagree | Somewhat agree | Strongly agree | Not Applicable |
| --- | --- | --- | --- | --- | --- | --- |

|  | 1 | 2 | 3 | 4 | 5 |
| --- | --- | --- | --- | --- | --- |

| The research evidence supporting the recommendations fits with my understanding of early diagnosis of cerebral palsy (CP), intervention and surveillance | 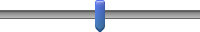 |
| --- | --- |
| The research evidence is useful in thinking about the early diagnosis of cerebral palsy (CP), intervention and surveillance | 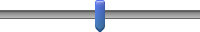 |
| I am clear about the key messages for implementing the best practice recommendations | 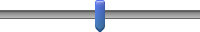 |
| There is consensus among my colleagues about the usefulness of the research evidence about early diagnosis of cerebral palsy (CP), intervention and surveillance | 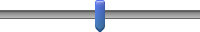 |

This section is if you would like to add any additional comments relating to the Research Evidence supporting the newly released best practice recommendations for the early diagnosis of cerebral palsy (CP), intervention and surveillance.

|  |
| --- |

Q8 This section is about the value of Clinical Experience as additional evidence in the early diagnosis of cerebral palsy (CP), intervention and surveillance

To what extent do you agree with the following statements.

|  | Strongly disagree | Somewhat disagree | Neither agree nor disagree | Somewhat agree | Strongly agree | Not Applicable |
| --- | --- | --- | --- | --- | --- | --- |

|  | 1 | 2 | 3 | 4 | 5 |
| --- | --- | --- | --- | --- | --- |

| I am able to draw on my own clinical experience as additional evidence in the early diagnosis of cerebral palsy (CP), intervention and surveillance | 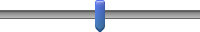 |
| --- | --- |
| I have shared and critically reviewed my clinical experience with knowledgeable colleagues outside of my (clinical) workplace | 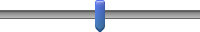 |
| There is consensus of clinical experience about the early diagnosis of cerebral palsy (CP), intervention and surveillance | 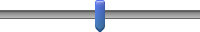 |
| Clinical experience is important in the implementation of the best practice recommendations | 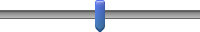 |
| Clinical experience as evidence is useful in thinking about implementing these recommendations | 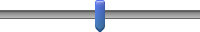 |

This section is if you would like to add any additional comments relating to Clinical Experience as additional evidence in the early diagnosis of cerebral palsy (CP), intervention and surveillance

|  |
| --- |

Q9 This section is about the Evidence from the **Family/Whānau Experience**

To what extent do you agree with the following statements.

|  | Strongly disagree | Somewhat disagree | Neither agree nor disagree | Somewhat agree | Strongly agree | Not Applicable |
| --- | --- | --- | --- | --- | --- | --- |

|  | 1 | 2 | 3 | 4 | 5 |
| --- | --- | --- | --- | --- | --- |

| We routinely (and systematically) collect family/whānau experiences about early diagnosis of cerebral palsy (CP), intervention and surveillance | 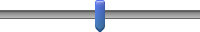 |
| --- | --- |
| Family/whānau experiences are seen to be an important source of evidence in implementing these recommendations | 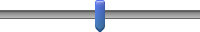 |
| I value family/whānau experiences as evidence | 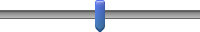 |
| Family/whānau experiences fit my understanding of the early diagnosis of cerebral palsy (CP), intervention and surveillance | 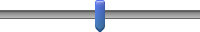 |
| There is consensus among my colleagues about the usefulness of family/whānau experiences in the early diagnosis of cerebral palsy (CP), intervention and surveillance | 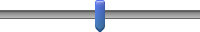 |
| Early diagnosis of Cerebral Palsy fits within the Family/ Whānau Integrated Care Model | 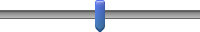 |

This section is if you would like to add any additional comments relating the Evidence from the Family/Whānau Experience

|  |
| --- |

# Block 3: Context

The ADHB NICU and community health services who care for former NICU babies in the first 6 months is the environment where the best practice guideline will be implemented

Q10 **Context –** This section is about the environment (NICU and community) in which the best practice recommendations will be implemented (Link to best practice recommendations**)**

  To what extent do you agree with the following statements.

|  | Strongly disagree | Somewhat disagree | Neither agree nor disagree | Somewhat agree | Strongly agree | Not Applicable |
| --- | --- | --- | --- | --- | --- | --- |

|  | 1 | 2 | 3 | 4 | 5 |
| --- | --- | --- | --- | --- | --- |

| The physical location is suitable for implementation the recommendations | 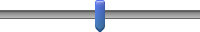 |
| --- | --- |
| There are sufficient human resources to implement the recommendations successfully | 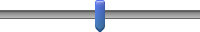 |
| There are sufficient financial resources to implement the recommendations successfully | 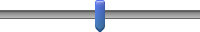 |
| There is the right equipment to implement the recommendations successfully | 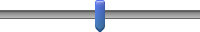 |

| There is the right IT support to implement the recommendations successfully | 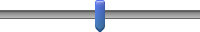 |
| --- | --- |
| I have access to the appropriate/useful professional networks to help me implement the recommendations successfully | 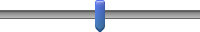 |
| The recommendations fit with the strategic intent and goals of our organization | 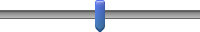 |
| Decision-making processes in the organization are clear to me | 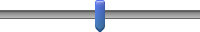 |

This section is if you would like to add any additional comments about the **environment (NICU or community)**in which the best practice recommendations will be implemented

|  |
| --- |

Q11 **Context – this section is about the culture of the environment (NICU or community) for implementing the best practice recommendations**

 
To what extent do you agree with the following statements.

|  | Strongly disagree | Somewhat disagree | Neither agree nor disagree | Somewhat agree | Strongly agree | Not Applicable |
| --- | --- | --- | --- | --- | --- | --- |

|  | 1 | 2 | 3 | 4 | 5 |
| --- | --- | --- | --- | --- | --- |

| I have the power and authority to contribute to the implementation of the recommendations | 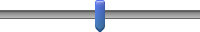 |
| --- | --- |
| I have access to the appropriate skills and knowledge to the recommendations | 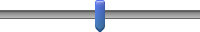 |
| This organisation values innovation | 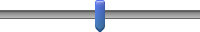 |
| This organisation values people who innovate | 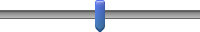 |
| This organisation values staff as individuals | 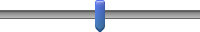 |
| This organisation values the uniqueness of each baby and their family/whānau situation | 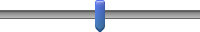 |
| This organisation values open communication and dialogue | 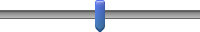 |
| This organisation values relationships with others | 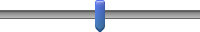 |
| This organisation values team work () | 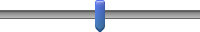 |
| This organisation values power and authority | 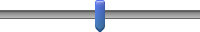 |
| I feel there is open communication and dialogue within my immediate work place | 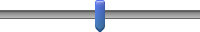 |
| I value open communication and dialogue | 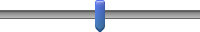 |
| This organization values collaborative working partnerships | 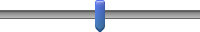 |
| I feel there is collaborative working partnerships in the wider organization | 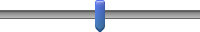 |

| I feel there is collaborative working partnerships within my immediate work place | 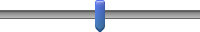 |
| --- | --- |
| I value collaborative working partnerships | 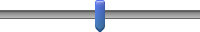 |
| I actively participate in the implementation of best practice/ new evidence | 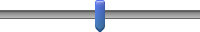 |
| Our unit is supportive of implementing best practice/new evidence | 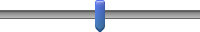 |
| I prefer to keep things as they are | 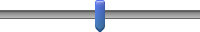 |

This section is if you would like to add any additional comments about the **culture of the environment (NICU or community)** for implementing the best practice recommendations

|  |
| --- |

Q12 **Context – this section is about the leadership operating in the environment into which the best practice recommendations will be implemented**

 
To what extent do you agree with the following statements.

|  | Strongly disagree | Somewhat disagree | Neither agree nor disagree | Somewhat agree | Strongly agree | Not Applicable |
| --- | --- | --- | --- | --- | --- | --- |

|  | 1 | 2 | 3 | 4 | 5 |
| --- | --- | --- | --- | --- | --- |

| I am clear about my role within the team | 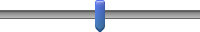 |
| --- | --- |
| I am clear what my role is in the implementation of these best practice recommendations | 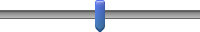 |
| I have been involved in determining how these best practice recommendations are going to be implemented | 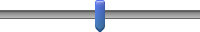 |
| I am clear about the lines of accountability are in terms of my role in implementing these best practice recommendations | 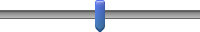 |
| I have been able to develop new skills through this process | 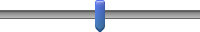 |
| I feel that l have learnt new skills and competencies | 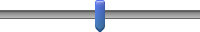 |

This section is if you would like to add any additional comments about the **leadership in the environment (NICU or community)** into which the best practice recommendations will be implemented.

|  |
| --- |

Q13 Context – this section is about the evaluation processes in place in the environment into which the best practice recommendations will be implemented (ADHB NICU and health services involved in the care of former NICU babies in the first 6 months)

 
To what extent do you agree with the following statements.

|  | Strongly disagree | Somewhat disagree | Neither agree nor disagree | Somewhat agree | Strongly agree | Not Applicable |
| --- | --- | --- | --- | --- | --- | --- |

|  | 1 | 2 | 3 | 4 | 5 |
| --- | --- | --- | --- | --- | --- |

| We have routine mechanisms in place to collect data on Individual performance (e.g., appraisal, clinical supervision, 360° feedback) | 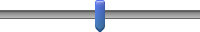 |
| --- | --- |
| We have routine mechanisms in place to collect data on Team performance (e.g., audit and feedback, patient feedback, 360° feedback) | 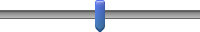 |
| We have routine mechanisms in place to collect data on System performance (e.g., audit and feedback, formal inspections, economic data) | 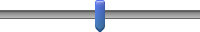 |
| Multiple sources of evaluation are used routinely in my workplace | 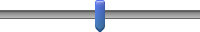 |
| This type of evaluative information is routinely used to improve and change practice | 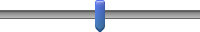 |
| The external data we collect is used to inform and improve our everyday practice. | 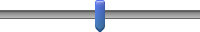 |

This section is if you would like to add any additional comments about the **evaluation processes in place in the environment** into which the best practice recommendations will be implemented

|  |
| --- |

# BLOCK 4: Practical considerations

|  | Strongly disagree | Somewhat disagree | Neither agree nor disagree | Somewhat agree | Strongly agree | Not Applicable |
| --- | --- | --- | --- | --- | --- | --- |

|  | 0 | 1 | 2 | 3 | 4 | 5 |
| --- | --- | --- | --- | --- | --- | --- |

| FTE will need to be added for coordinating assessments for babies on this pathway | 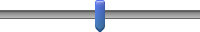 |
| --- | --- |
| FTE will need to be added for interpretation of results and communication to families for babies on this pathway | 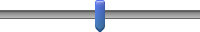 |
| I have an understanding of the assessments used in this pathway | 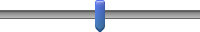 |

Approximately 90 babies per year will meet the criteria for the cerebral palsy screening pathway within the ADHB. Their assessments may involve MRI, Hammersmith Infant Neurological Exam (HINE), and Prechtl's General Movements Assessment (GMA).

|  | Strongly Disagree | Somewhat Disagree | Neither Disagree nor Agree | Somewhat Agree | Strongly Agree | Not Applicable |
| --- | --- | --- | --- | --- | --- | --- |

|  | 0 | 1 | 3 | 4 | 5 |
| --- | --- | --- | --- | --- | --- |

| MRI at term on all babies on the pathway is feasible. (Around 90 MRI/year) | 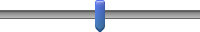 |
| --- | --- |
| MRI at term on all babies on the pathway is a justified use of MRI resources. | 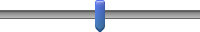 |
| I understand the value of MRI in the early diagnosis of cerebral palsy. | 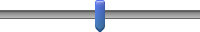 |
| Hammersmith Infant Neurological Exam (HINE) at 12-14 weeks CGA on all babies on the pathway is feasible. | 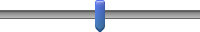 |
| I understand the value of the HINE in the early diagnosis of cerebral palsy. | 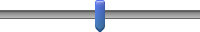 |
| Filming General Movements Assessment (GMA) on all babies in this pathway at 32-35 weeks, term and 12-14 weeks CGA is feasible | 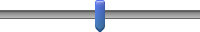 |
| Having trained assessors to review and interpret GMA videos for all babies on this pathway is feasible. (around 270 videos/year) | 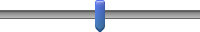 |
| I understand the value of GMA in the early diagnosis of cerebral palsy. | 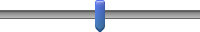 |

Q16 Hammersmith Infant Neurological Exam (HINE)

- I am unfamiliar with the HINE
- I am familiar with the HINE but do not routinely use it
- I routinely use the HINE
- Other ________________________________________________

Q17 Prechtl's General Movement's Assessment (GMA) (tick all that apply)

- I am unfamiliar with the GMA
- I am familiar with the GMA but do not routinely use it
- I routinely use the GMA in my practice
- I have experience filming GMA videos
- I am trained in interpreting GMA videos

Q18 Who is the best person to film the General Movements Assessment (GMA)? (tick all that apply)

- Registrar/ NP/ NSANP
- Neonatologist/ Fellow
- Physio/Occupational therapist
- Bedside nurse
- Senior nurse
- Parents
- Other ________________________________________________

Q19 Which professional group should be trained to assess the General Movements Assessment (i.e to review/score the video)? (you may tick more than one)

- Neonatologist/ Fellow
- Registrar/ NSANP/NP
- Physio/Occupational therapist/Neurodevelopmental team
- A multidisciplinary team who are interested in doing the training
- Senior nurse
- Assessors should be external to the DHB
- Other ________________________________________________

Q20. Who should be responsible for collating the results from the different assessments?

Q21. Thank you. Do you have any further comments?
