# Supplementary material for: A prospective assessment of readiness to implement an early detection of cerebral palsy pathway in a neonatal intensive care setting using the PARIHS framework
Source: Implement Sci Commun. 2024 Apr 23;5:46. doi: 10.1186/s43058-024-00581-0 (PMC11036598; doi:10.1186/s43058-024-00581-0)
Supplement: Supplementary file 3 — Additional file 3. Focus group structure. [file 43058_2024_581_MOESM3_ESM.docx]

Supplementary 3: Focus group structure

Semi-structured discussions with focus groups (grouped according to profession to minimise potential power imbalance) took place in a private meeting room within the workplace (during work hours, approximately 1 hour) in groups of 2-5. Two participants chose to interview individually via zoom. Discussions were facilitated by a female neonatal nurse (AM) familiar with the topic and the study setting. The gender balance was mostly female, there was one male participant in the focus groups. The interviewer was known to some of the participants but not all and was not in a supervisory or management role for any of the participants nor were they involved in the development of the early detection of cerebral palsy (CP) pathway being discussed

The session began with karakia (blessing/intention) and introducing ourselves. Study information provided again along with a question time before written consent was obtained. An overview of the early detection of CP pathway was then provided, followed by discussion points related to the PARIHS constructs *Evidence, Context,* ideas about how to facilitate the implementation of the pathway, and what would work practically within the NICU context. Questions were based around the flow chart below with further information gained by the interviewer seeking clarification or asking for more details e.g., “tell me about...”, “what was the impact of …?”, “what do you think needs to happen …”. Not every question was asked in every group, it was a guide for the interviewer to stimulate discussion, however the participants were encouraged to focus on the areas that they felt they wanted to talk about.

Table: Focus group question structure

| **Evidence** | *Research, Clinical experience, Whānau/family experience* | What is your understanding of the impact on babies and whānau of delayed diagnosis of CP or high risk of CP? (Please note that going forward diagnosis refers to both a diagnosis of CP and a diagnosis of High Risk of CP) |
| --- | --- | --- |
|  |  |  |
|  |  | What is your knowledge of early treatment options for CP? |
|  |  | Do you feel there is consensus around the need for early diagnosis of CP across teams? How does this impact care? |
|  |  | How comfortable do you feel about a diagnosis of CP or high risk of CP for babies under 6 months. What are the pros and cons from your perspective? |
|  |  | What is the impact of a delayed diagnosis (positive or negative)? |
|  |  | How urgent is it to implement this pathway? |
| **Context** | *Resources - knowledge, leadership, capacity, training, staffing, access to MRI*  *Culture - ADHB values* | What are our strengths? |
|  |  | What are things that are already in place that will make implementation simple? |
|  |  | What are the barriers? |
|  |  | What changes need to happen to allow culture/ leadership/ systems that encourage transformational change? |
|  |  | How will we evaluate if we are meeting the needs of babies with cerebral palsy?  What processes will there be for feedback?  Is this feedback valued by the unit/ by you? |
|  |  | What has worked well for this NICU, for previous policy changes? |
| **Facilitation preparation** | Practical questions around how assessments would work, e.g. what technology, training, resources are needed? | MRI availability/cost  HINE training, who would do it?  GM inpatient/community  (paperwork/consent/filming/IT storage/assessment/ reporting  Collating and communicating with family  Followup support and treatment for CP or high risk of CP diagnosis |
| **MRI** | *Some babies on pathway will already have MRI e.g. HIE* | How do you see these recommendations impacting current MRI demand? |
| **HINE** | *In this pathway HINE is due around 12 weeks CGA* | More for groups who see baby in clinic or community |
|  | *In the survey 76% felt it was feasible to do HINE on babies at 12-14 weeks either by Community VT or by SMO in clinic* | Do you use HINE currently?  If yes when? |
|  |  | How easy would it be to embed HINE into follow up clinic/ community care? What is needed to do this? |
| **GM** | *GM is due Preterm (32-35w), Term, 12-14weeks. Show example paperwork and clamp ipad holder* | *Have a look at the paperwork examples and invite people to write on feedback* |
|  |  | How do you envisage GM’s filming and assessment would work in NICU and in the community?  Who will film?/where will they upload it to?/ who will assess |
| **General** |  | What is the best way to support people to implement this guideline? |
|  |  | Tell me about any concerns you have about collating and communicating with family |
|  |  | What is helpful in everyday practice? |
|  |  | What would you name the pathway? |
|  |  | What would change for the services provided by the community with a CP or high risk of CP diagnosis? |
