# Supplementary material for: A prospective assessment of readiness to implement an early detection of cerebral palsy pathway in a neonatal intensive care setting using the PARIHS framework
Source: Implement Sci Commun. 2024 Apr 23;5:46. doi: 10.1186/s43058-024-00581-0 (PMC11036598; doi:10.1186/s43058-024-00581-0)
Supplement: Supplementary file 4 — Additional file 4. Code book. [file 43058_2024_581_MOESM4_ESM.docx]

Supplementary 4_Codebook

Early Identification of Cerebral Palsy Pathway Implementation Focus Groups

Codes

| Name | Description | Files | References |
| --- | --- | --- | --- |
| Context | Comments relating to the context of where the guideline will be implemented | 1 | 2 |
| ADHB NICU specific | Comments relating specifically to the ADHB NICU context | 5 | 17 |
| An Enabler | Enabling factors for implementation of guideline specific to ADHB NICU context | 2 | 5 |
| Barrier | Barriers to implementation of guideline specific to ADHB NICU context | 4 | 11 |
| culture | Comments relating to workplace culture at ADHB | 3 | 8 |
| Barrier | Comments relating to workplace culture at ADHB which are a barrier to implementation of guideline | 2 | 5 |
| Enabler | Comments relating to workplace culture at ADHB which are an enabler to implementation of guideline | 3 | 6 |
| Naming the pathway | Comments relating to potential names for the pathway | 8 | 44 |
| Barrier | Comments relating to perceived barriers to naming the pathway | 3 | 5 |
| Enabler | Comments that are enablers for naming the pathway | 5 | 16 |
| Community specific | Comments relating to the guideline in outpatient and community care settings | 7 | 34 |
| Barrier | Barriers to implementation of the guideline in community context | 6 | 28 |
| Enabler | Enablers to implementation of the guideline in community context | 5 | 16 |
| Equity | Comments about how the guideline impacts equitable care both regionally, socially and culturally | 7 | 42 |
| Barrier | Aspects of guideline which could cause inequality or barriers to equitable implementation | 7 | 42 |
| Enabler | Aspects of guideline which could increase equity of care and enablers to equitable implementation | 3 | 4 |
| health system | Comments relating to context of how the health system (te whatu ora/dhb) works and how that might impact implementation of guideline | 8 | 24 |
| Barrier | Comments relating to barriers of the health system impacting implementation | 7 | 14 |
| Enabler | Comments relating to enabling aspects of the health system impacting implementation | 3 | 4 |
| Knowledge and skill of staff | Comments relating to knowledge and skills of staff. Eg required knowledge and training to implement guideline | 8 | 42 |
| Barrier | Comments relating to barriers for staff to access appropriate information or training in order to implement guideline | 8 | 33 |
| Enabler | Comments relating to staff knowledge and training that will enable implementation | 6 | 19 |
| GM | Comments specific to general movements | 8 | 66 |
| Barrier | Comments specific to general movements barriers | 8 | 47 |
| Enabler | Comments specific to general movements enablers | 7 | 20 |
| HINE | Comments specific to Hammersmith Infant Neurological Exam (HINE) | 6 | 25 |
| Barrier | Comments specific to HINE barriers to implementation | 6 | 16 |
| Enabler | Comments specific to HINE enablers to implementation | 4 | 14 |
| MRI | Comments specific to MRI | 7 | 36 |
| Barrier | Comments specific to MRI barriers | 7 | 37 |
| Enabler | Comments specific to MRI enablers | 5 | 7 |
| Leadership | Comments relating to leadership (including champions) and how it might affect the implementation of the guideline | 7 | 18 |
| Barrier | Contextual barriers relating to leadership including champions | 5 | 10 |
| Enabler | Contextual enablers relating to leadership including champions | 5 | 12 |
| Resources | Comments relating to resources required or available for implementation of guideline. General “Resources” code could be a combo of different resources. More specific codes below. | 8 | 69 |
| Barrier | Comments relating to barrier to implementation due to resource (see above) | 8 | 59 |
| Enabler | Comments relating to enabler to implementation due to resource (see above) | 7 | 24 |
| Personal | Comments specific to personal resources (which includes FTE, time and staffing) and how this affects implementation of guideline | 8 | 78 |
| Barrier | Comments relating to personal resources (see above) that are a barrier to implementation | 8 | 50 |
| Enabler | Comments relating to personal resources (see above) that are an enabler to implementation | 8 | 20 |
| systems | Comments specific to systems resources (eg booking systems, clinic set up, IT etc) and how this affects implementation of guideline | 8 | 51 |
| Barrier | Comments relating to systems resources (see above) that are a barrier to implementation | 8 | 40 |
| Enabler | Comments relating to systems resources (see above) that are an enabler to implementation | 4 | 9 |
| Evaluation | Relates to how implementation would be monitored and evaluated to assess if it is working as intended | 5 | 19 |
| Barrier | Barriers to meaningful evaluation (as above) of the guideline implementation | 3 | 14 |
| Enabler | Enablers to meaningful evaluation (as above) of guideline implementation | 1 | 1 |
| Evidence | Evidence includes the guideline and research behind the guideline as well as clinical experience, family experience and local data | 0 | 0 |
| Clinical Experience | Clinical experience valued as evidence from staffs’ point of view | 8 | 71 |
| Barrier | Clinical experience which indicates a barrier to implementation | 8 | 52 |
| Enabler | Clinical experience which indicates an enabler to implementation | 8 | 23 |
| Family Experience | Family experience valued as evidence from staffs point of view | 8 | 95 |
| Barrier | Family experience valued as evidence which is a barrier to implementation of guideline | 8 | 61 |
| Enabler | Family experience valued as evidence which is an enabler to implementation of guideline | 8 | 35 |
| local data | Local information (Auckland NICU and Community) as evidence | 7 | 16 |
| Barrier | Comments relate to local information as evidence that is a barrier to implementation | 3 | 9 |
| Enabler | Comments relate to local information as evidence that is an enabler to implementation | 5 | 6 |
| research | Includes the guideline as a piece of evidence and the research behind the guideline | 3 | 10 |
| an Enabler Research | Indicated approval of guideline recommendations or evidence base | 0 | 0 |
| Barrier Research | Indicated controversy or doubt about guideline recommendations or evidence base | 0 | 0 |
| consensus | Comments relating to consensus about aspects of the guideline or evidence base | 8 | 26 |
| Barrier | Indicated lack of consensus about aspects of guideline or evidence base | 6 | 15 |
| Enabler | Indicated consensus about aspects of guideline or evidence base | 5 | 11 |
| evidence for treatment options | Evidence and research regarding treatment options for cerebral palsy | 4 | 23 |
| Barrier | Indicated concern or disagreement about treatment options or aspects of treatment options for cp | 4 | 16 |
| Enabler | Indicated agreement for treatment options or aspects of treatment options for cp | 2 | 4 |
| Importance of evidence | Value staff placed on the guideline and evidence behind the guideline | 7 | 91 |
| Barrier | Value placed on the guideline which is a barrier to implementation | 7 | 43 |
| Enabler | Value placed on the guideline which is an enabler to implementation | 7 | 37 |
| Ways of implementing | Comments that are ideas for the way guideline could be implemented | 5 | 13 |
| Great Quotes |  | 8 | 48 |
